# Effects of Dietary *β*-Carotene Supplementation on Growth Performance, Biochemical Indices, Hemato-Immunological Parameters, and Physio-Metabolic Responses of the Oriental River Prawn (*Macrobrachium nipponense*)

**DOI:** 10.1155/anu/5184405

**Published:** 2025-03-25

**Authors:** Mohammad Ettefaghdoost, Hamid Navirian, Hossein Haghighi

**Affiliations:** Fisheries Department, Faculty of Natural Resources, University of Guilan, Sowmeh Sara, Guilan, Iran

**Keywords:** *β*-carotene, antioxidant, gene expression, innate immunity, oriental river prawn

## Abstract

An 8-week feeding experiment was carried out to determine the influence of *β*-carotene intake on the growth, biochemical, and immunological responses of *Macrobrachium nipponense*. Five distinct formulated nutritional regimens were fed to prawns with an average weight of 1.37 ± 0.09 g, each containing varying levels of *β*-carotene, including 0 (control), 50, 100, 150, and 200 mg/kg. According to the results, the growth metrics were markedly increased by the various levels of *β*-carotene (*p* < 0.05); however, the hepatosomatic index (HSI) remained statistically unchanged compared to the control treatment (*p* > 0.05). Despite that, a reduction was observed in most hemolymph biochemical indicators, including triglycerides, urea, creatinine, and uric acid (*p* < 0.05). Conversely, high-density lipoprotein (HDL) and low-density lipoprotein (LDL) levels demonstrated a notable increase in comparison to the control group (*p* < 0.05). Moreover, the levels of calcium, phosphorus, and cholesterol did not exhibit any noteworthy differences (*p* > 0.05). The administration of *β*-carotene resulted in a significant reduction of hemato-immune indices, including lactate dehydrogenase (LDH), aspartate aminotransferase (AST), alanine aminotransferase (ALT), and cortisol (CORT) levels. Conversely, there was a notable increase in the levels of lysozyme (LYZ), albumin (ALB), phenoloxidase (PO), total protein (TP), hyaline cells (HCs), granular cells (GCs), semi-GCs (SGCs), and total hemocyte count (THC) within the *β*-carotene treatment groups (*p* < 0.05). Furthermore, the majority of the hepatopancreatic antioxidant parameters, including malondialdehyde (MDA), catalase (CAT), and superoxide dismutase (SOD), exhibited a significant decrease in response to *β*-carotene treatment. However, the total antioxidant capacity (T-AOC) demonstrated a marked increase when compared to the control treatment (*p* < 0.05). Nonetheless, the levels of glutathione peroxidase (GPx) and alkaline phosphatase (AKP) did not exhibit any significant changes by these experimental treatments (*p* > 0.05). The research revealed that elevated dietary levels of *β*-carotene, specifically at 200 mg/kg, significantly increased digestive enzyme activities, total carotenoid content (TCC), and body chemical composition, including crude protein and crude lipid levels (*p* < 0.05). Despite the *β*-carotene supplementation, the populations of lactic acid bacteria (LAB) and ash content remained unaffected (*p* > 0.05). Notably, an increase in the levels of *β*-carotene corresponded with a significant elevation in the expression of genes related to growth and immunity (*p* < 0.05). An elevation in dietary *β*-carotene significantly increased polyunsaturated fatty acids (PUFAs), monounsaturated fatty acids (MUFAs), and essential amino acids (EAAs) compared to the control group (*p* < 0.05). Eventually, the present research results indicate that the incorporation of 200 mg/kg *β*-carotene pigment into dietary of the oriental river prawn positively influences the growth indices, hematological, immunological, and metabolic responses of this prawn species.

## 1. Introduction

Growth performance enhancement has been identified as one of the primary topics in aquaculture research, and it has also been proposed that the efficiency of reproduction in prawns is strongly associated with the development of diets and nutritional supplementation. The significance of carotenoid pigments in aquaculture nutrition and reproduction has stimulated an increased interest in aquaculture products due to their contributions to optimal growth, nutritional performance, immunological responses, enhancing market appeal, and greater resistance to adverse environmental conditions [1, 2]. Currently, natural carotenoids are preferred in aquaculture because they are more available and economically viable compared to synthetic ones [3, 4]. Under these natural carotenoids, *β*-carotene, derived from microalgae, carrots, and some vegetables, has been identified as an important pigment [5, 6]. In addition to its pigmentation characteristics, *β*-carotene demonstrates the highest retinol function among all carotenoid pigments. The molecular configuration of *β*-carotene indicates its potential for enzymatic cleavage into two molecules of retinol via a reduction process. Furthermore, *β*-carotene has been shown to effectively quench singlet oxygen, thereby, preventing lipid peroxidation. This property underscores its significant role as both an antioxidant and an immunostimulant [6–9].

The oriental river prawn, scientifically known as *Macrobrachium nipponense*, is considered among the top valuable and economically necessary freshwater aquatic species belonging to the family Palaemonidae [10, 11]. In comparison with the giant river prawn, *Macrobrachium rosenbergii*, the mentioned species had roughly 20% higher growth and a better survival rate (SR) at its larval stages. Furthermore, it illustrates an ability to withstand low temperatures throughout the winter season [12, 13]. The oriental river prawn was distributed among many countries owing to its several valuablefeatures, which include its ability to survive various environmental temperatures, especially low-temperature levels, increasing the SR. In addition, it shows better growth in natural environments and allows different types of rearing techniques, including pools, semi-intensive or intensive aquaculture systems, cages, and paddy fields in polyculture conditions. This species is notable for its reproduction efficiency and high economic value due to the fact that its breeding cycle takes approximately 80 days, thus, relatively shorter than in other cultivated prawn species [14–17]. With such a high economic perspective and the aforementioned characteristics, the prawn is regarded as one of the most in-demand aquaculture species for brackish and freshwater environments [18, 19]. Research indicates that *M. nipponense* demonstrates effective utilization of formulated diets, and its growth and immunity under culture conditions are notably enhanced when provided with an appropriate dietary formulation [20–22]. Thus, the focus should be on challenges associated with the aquaculture of this prawn species, particularly with regard to the implementation of specifically formulated diets capable of enhancing its growth in captivity conditions. Hence, a wide range of research studies has been carried out in this field concerning aquatic species, such as Jiang et al. [23] conducted a study examining the influence of different *β*-carotene levels on preadult male Chinese mitten crab (*Eriocheir sinensis*); Fawzy et al. [24] studied the impact of varying canthaxanthin dietary levels on the growth, antioxidant status, and immune-physiological responses of white shrimp (*Litopenaeus vannamei*); and the study conducted by Cheng and Wu [25] investigated the influence of dietary astaxanthin on the growth performance and immune response of the red swamp crayfish (*Procambarus clarkii*).

Our study posits that, considering the aforementioned outlined attributes of oriental river prawn aquaculture and the significant role of incorporating *β*-carotene into their diets to enhance growth performance and immune responses, there is an urgent necessity to promote awareness about dietary supplements that include these pigments. This knowledge is crucial for the formulation of tailored and cost-effective nutritional plans specifically designed for this species, with the aim of improving breeding conditions and mitigating challenges associated with pathogens. Our research enhances the comprehension of carotenoid application in aquatic nutrition and its significance for physiological and immunological functions. A principal aim of this investigation is to assess the use of *β*-carotene as a natural carotenoid pigment within dietary compositions, positioning it as a viable alternative to synthetic pigments for commercial purposes. Additionally, the findings of this research may establish a foundation for the ideal diet composition for diverse freshwater prawn species and other economically important crustaceans. Accordingly, the current research focused on assessing the *β*-carotene pigment influence on growth metrics, biochemical composition, immunological indices, physio-metabolic responses, and expression of growth and immune-related genes in the oriental river prawn, which is noted for its excellent potential for economic production and breeding in a variety of freshwater environments.

## 2. Materials and Methods

### 2.1. Conditions for Prawn Cultivation

The feeding trial was carried out over a period of 8 weeks at the University of Guilan, as well as at the Fishland Aquarium located in Rasht city, Gīlān Province, Iran. The examined prawns in the current study were captured using trap nets (Damidel, Model 32 in ×10 in Folded, Victoria, Seychelles), exhibiting a weight range and total length of 1–1.5 g and 5 cm, respectively. The specimens were obtained from the Siah Darvishan River (lat 25°37′ N, long 49°30′ E, alt -15 AMSL, Tulem District, Gīlān, Iran). Eventually, the prawns were transported to the experimental site for further analysis. The prawns were maintained in a 1000-L tank (Reevoo Industrial Fabric Co., Ltd., Model PVC 1000D*⁣*^*∗*^1000D 23*⁣*^*∗*^23, Zhejiang, China) for a duration of 14 days to facilitate acclimatization to the physicochemical parameters of the water. Throughout this acclimation phase, the prawns were provided with ad libitum access to a basal diet formulated for oriental river prawns, which was shaped in a 1 mm crumble form. The composition of this diet included moisture, protein, lipid, and ash percentages of 10%, 45%, 5%, and 14%, respectively [26, 27]. Following completion of the adaptation phase, the prawns were weighed employing an electronic balance (Sartorius, Model GM612, Göttingen, Germany) and measured with a digital caliper (iGAGING, Model 100-030N, California, USA). Prawns exhibiting an average initial weight of 1.37 ± 0.09 g and a mean total length of 5.07 ± 0.20 cm were chosen and subsequently allocated at random into 15 glass aquariums (Fishland Aquarium Products, Model 80 length ×35 width ×45 depth cm/8 mm, Rasht, Iran), with each aquarium containing 25 prawns. Each experimental glass aquarium was loaded with 90 L of dechlorinated purified water that had been aerated for a period of 24 h prior to the feeding trial. Continuous aeration in the aquariums was maintained throughout the experimental period via an air–stone (Mahiran, Model HJ-110, Tehran, Iran) connected to a central aerator (Hailea, Model ACO-500, Guangdong, China). Daily water changes consisted of replacing 1/3rd of the aquarium capacity before feeding each day, and whole-water replacement was done with dechlorinated water during the biometry process. Water sampling was conducted prior to the initiation of the feeding process, and the assessment of water quality parameters was carried out in accordance with the methodology outlined by the American Public Health Association [28]. Environmental conditions in the culture were scheduled at a 12 h light and 12 h dark photoperiod (12L:12D) using a fluorescent lamp (Dymax, Model T5, Changi, Singapore).

### 2.2. Preparation of Diet and Experimental System

The feeding trial dietary treatments were formulated based on the fundamental diets of the *M. nipponense*, utilizing software (LINGO Version 20.0, Illinois, USA) for the adjustments. The preparation of each diet commenced with the complete pulverization of the original ingredients using a grinder (Bariton, Model BFC-18500GX, Ontario, Canada), and thereafter, sieved through a 100 μm mesh (DG Scientific Products, Model ASTM E11 standard, Tehran, Iran) to achieve a homogeneous sample, followed by the removal of all impurities.

The preliminary materials necessary for the formulated dietary treatments were accurately measured utilizing an electronic balance and total constituents were thoroughly combined (Waring, Model 24CB9EC, Pennsylvania, USA) for a duration of 15–20 min. Afterward, deionized water (Ghatran Shimi, Catalog No. 25100, Tehran, Iran), constituting approximately a 3:10 ratio of the dry substance, was incorporated into the mixture. The resultant mixture was then entered into an extruder (MIKIM, Model DF-70, Tianjin, China), where exclusive feed pellets of approximately 1 mm in diameter were made.

In the final stage, *β*-carotene powder (10% CWS/S, DSM Nutritional Products, Basel, Switzerland) was dissolved in distilled water using a magnetic stirrer (Mtops, Model HSD-180, Seoul, South Korea) and subsequently sprayed onto the experimental diets. Following the drying process, the samples were preserved at −16°C in a freezer (TAT, Model FM25100, Tehran, Iran). Given the sensitivity of *β*-carotene to environmental fluctuations, the daily dietary samples were preserved in opaque polymeric receptacles within a laboratory fridge (TAT, Model 300-Lit, Tehran, Iran) maintained at approximately 4°C.

The feeding trial treatments comprised five distinct diets and three replicates, incorporating varying control levels—specifically, a control group without *β*-carotene pigment and groups supplemented with *β*-carotene at dietary amounts of 50, 100, 150, and 200 mg/kg. These diets were administered to the prawns over a period of 56 days. Feeding was conducted manually four times daily (at 08:00, 12:00, 16:00, and 20:00 h) at a rate equivalent to 3% of the prawn's biomass for each trial replication [26, 27, 29]. The proximate analysis of the experimental diets was conducted in accordance with the AOAC [30] methodology, while the total carotenoid content (TCC) of the diets utilized in this study was assessed through spectrophotometric analysis [23, 31].  Table 1 provides a comprehensive overview of the composition and approximate analysis of the trial diets employed during the cultivation period of *M. nipponense* in this research.

### 2.3. Assessment of Water Physicochemical Quality Parameters

The physicochemical quality parameters of water, including temperature and dissolved oxygen, were monitored daily. Concurrently, additional parameters such as total water hardness, phosphate, nitrate, ammonium, nitrite, and pH were assessed in conjunction with each biometry evaluation. The temperature was monitored using a TFA Dostmann GmbH device (Model 30.1040, Wertheim, Germany), and dissolved oxygen levels were determined with a Hannah instrument (Model HI98198-opdo, Virginia, USA). Furthermore, pH, ammonium, nitrite, nitrate, and phosphate concentrations were quantified using Milwaukee equipment (Rocky Mount, USA), while total water hardness was assessed with a hardness tester from HM Digital Inc. (Model COM-100, Carson, USA) [28].

### 2.4. Assessment of Growth Metrics

Initially, feeding was halted for a duration of 24 h after the completion of the trial period, during which time the prawns from each individual replication were subjected to measurement. Various growth performance metrics were determined through calculations employing the following formulas [32–34]:

Weight gain (WG; g) = final weight of prawns (g) − initial weight of prawns (g)

WG (%) = 100 × [WG (g)/initial weight of prawns (g)]

Specific growth rate (SGR; %/day) = 100 × [(ln (final weight of prawns (g)) − ln (initial weight of prawns (g)))/trial duration (days)]

Feed conversion ratio (FCR) = feed given (g)/WG (g)

Hepatosomatic index (HSI; %) = (hepatopancreas weight of prawns (g)/weight of prawns (g))

SR (%) = 100 × (final count of prawns/initial count of prawns).

### 2.5. Hemolymph Sampling

Hemolymph specimens were collected from the ventral sinus of prawns with a 1 mL syringe (BD, Micro-Fine Plus, New Jersey, USA) that had been prerinsed with an equal-quantity dilution of Alsever's anticoagulant solution (CE-Immundiagnostika GmbH, Product Code AlseLö, Baden–Württemberg, Germany). Selected hemolymph samples were divided into aliquots and placed into microtubes (Explilab, Model Expl k2, Tehran, Iran), each containing an equal volume of 10% neutral buffered formalin solution (Neutron, Model NeutroFix 10, Product Code 1.3300, Tehran, Iran). The samples were then stored at a temperature of 4°C for the purpose of evaluating immunological parameters. Additional portions of the specimens were preserved in individual microtubes and stored in a deep freeze (Snijders Labs, Model VF120-86G, North Brabant, Netherlands) condition at −80°C for further assessment. Following the thawing process, the specimens underwent thorough homogenization utilizing a vortex (Cole–Parmer, Model V-200 Stuart, Illinois, USA) for a duration of 25–30 s. Eventually, the homogenized specimens were employed for analytical procedures [34–37].

### 2.6. Assessment of Hemato-Biochemical Indices

To assess the hemato-biochemical indices, a mixture of samples and Alsever's solution underwent centrifugation at 12,000 rpm and a temperature of 4°C for a period of 15 min, utilizing a centrifuge (Hettich GmbH, Model MIKRO 200R, Tuttlingen, Germany). Following centrifugation, the supernatant was carefully extracted using a micropipette (Corning, Model Lambda Plus, New York, USA) and subsequently loaded into 1.5 mL micro-centrifuge tubes. The biochemical indicators assessed included glucose, calcium, creatinine, urea, high-density lipoprotein (HDL), low-density lipoprotein (LDL), triglycerides, phosphorus, uric acid, and cholesterol. These measurements were conducted colorimetrically via an automated clinical chemistry analyzer (Biotecnica Instruments S.p.A., Model BT 4500, Rome, Italy) in conjunction with commercial kits (Pars Azmoon Co., Alborz, Iran) [34, 37, 38].

### 2.7. Assessment of Hemato-Immune Parameters

An evaluation of total hemocyte counts (THCs) was carried out using the Neubauer chamber in conjunction with a cover slip (HBG, Model Neubauer-Improved Bright lined Chamber, Berlin, Germany). The hemocytes were examined using a light microscope (A. Krüss Optronic GmbH, Model MBL2000-T-PL-PH, Hamburg, Germany) equipped with a 40x magnification objective lens. An approximate volume of 0.1 mL of the prawn's hemolymph specimen, combined with an anticoagulant solution, was applied to the microscope slide for the purpose of assessing the differential hemocyte counts (DHCs), which included semi-granular cells (SGCs), hyaline cells (HCs), and granular cells (GCs). After allowing the smears to air-dry, specimens were followed by a fixing process in 70% methanol (Merck Millipore, Model EMSURE CAS No. 67-56-1, Massachusetts, USA) for a duration of 10 min before undergoing the Giemsa staining procedure (Neutron, Catalog No. 1.2600, Tehran, Iran), each for 10 min. A total of 300 cells were enumerated and classified for each slide [34, 39–41]. The enzymatic activity of lysozyme (LYZ) was measured utilizing a turbidimetric assay, employing *Micrococcus luteus* (Sigma–Aldrich, ATCC No. 4698, Missouri, USA) as the substrate. A spectrophotometer (Agilent Technologies, Model BioTek 800TS, California, USA) was employed to record the absorbance at 450 nm. The activity of phenoloxidase (PO) was assessed utilizing spectrophotometric methods at a wavelength of 490 nm, which was predicated on the conversion of dopachrome. The enzymatic activities of lactate dehydrogenase (LDH), aspartate aminotransferase (AST), and alanine aminotransferase (ALT) were quantified at a wavelength of 340 nm, while the levels of alkaline phosphatase (AKP) were assessed at 405 nm. Concurrently, measurements of total protein (TP), cortisol (CORT), and albumin (ALB) were conducted utilizing commercial kits (Pars Azmoon Co., Alborz, Iran) [34, 38, 41].

### 2.8. Assessment of Antioxidant Activities

In order to assess antioxidant parameters, specimens of hepatopancreas from prawns were obtained, and thereafter, washed with distilled water at low temperatures. Following this, the samples were weighed and a Tris-EDTA buffer solution (Sigma-Aldrich, Product Code T 9285, Missouri, USA) with a 1:9 ratio was added. The samples were subjected to a homogenization process using a homogenizer (Wiggens GmbH, Model D-500 PRO, Düsseldorf, Germany) at a temperature of 4°C. Eventually, the specimens were undergoing a centrifugation process at 15,000 rpm for a period of 15 min; subsequently, the supernatant was meticulously extracted using a micropipette. The resulting specimens were then moved to sterilized micro-centrifuge tubes and subsequently stored at a temperature of −80°C for future analysis. The activity of antioxidants, specifically glutathione peroxidase (GPx), superoxide dismutase (SOD), malondialdehyde (MDA), total antioxidant capacity (T-AOC), and catalase (CAT), were assessed using colorimetric methods (Agilent Technologies, Model BioTek 800TS, California, USA) in conjunction with commercial kits (Teb Pazhouhan Razi, Code No. TPR-96, Tehran, Iran), following the protocols outlined by previous studies [34, 42, 43].

### 2.9. Assessment of Digestive Enzyme Activity

To assess the activities of digestive enzymes, the feeding procedure of prawns was stopped for a duration of 48 h prior to sampling, ensuring the complete evacuation of nutrients from the prawn's digestive tracts. The whole gastrointestinal tracts were obtained and maintained on ice at temperatures not exceeding 4°C to inhibit the digestive enzyme activities, after which any residual nutrients were eliminated. The specimens that were prepared underwent a washing process using ice-cold distilled water. After being measured using an electronic balance (Sartorius, Model BCE623-1S, Göttingen, Germany) with a precision of 0.001 g, the samples were combined with a buffer solution in a ratio of 1:9. The resulting mixture was then transferred into 2 mL micro-centrifuge tubes. The homogenization procedure was performed using a homogenizer for a duration of 30 s while kept on ice to maintain low temperatures. The resulting suspensions underwent centrifugation at a speed of 10,000 rpm for a duration of 10 min at a temperature of 4°C. The supernatant was carefully extracted using a micropipette and preserved in sterilized micro-centrifuge tubes at a deep freezing temperature −80°C. The enzymatic activity of digestive enzymes was quantified through spectrophotometric techniques utilizing commercial kits (Pars Azmoon Co., Alborz, Iran). Specifically, the activities of protease, amylase, and lipase were evaluated at wavelengths of 450, 540, and 405 nm, respectively. The results were expressed in terms of international units per milligram of protein [44–46].

### 2.10. Assessment of Intestinal Microflora

In order to evaluate the total bacterial count (TBC) and the count of lactic acid bacteria (LAB) within the intestines of experimental prawns, a period of 48 h without feeding was implemented prior to sampling. Prawns from the experimental replicates were chosen and subsequently subjected to anesthesia using ice powder. Following this process, the specimens were washed with demineralized water. The exoskeleton was then excised using a sterile scalpel and intestinal samples were collected with a bistoury knife. The specimens were measured after the thorough extraction of their internal components. The prepared samples were thoroughly homogenized for a period of 2 min at a ratio of 1:9 with a normal saline solution (Alkali Scientific, Model Cellpro—9 g NaCl per 100 mL, Florida, USA) employing a laboratory vortex mixer (Cleaver Scientific, Model 230V, Warwickshire, UK). Serial dilutions ranging from 10^−1^ to 10^−10^ were generated from the resulting solution. A volume of 100 μL from each dilution was then transferred using a micropipette, and the inoculation on trypticase soy agar (TSA) and de Man, Rogosa, and Sharpe (MRS) bacterial culture media (Quelab Laboratories Inc., Montréal, Canada) was done to ascertain TBC and LAB, respectively. The TSA and MRS plates were incubated for 48 h under aerobic (25°C) and anaerobic (30°C) circumstances, respectively. Following the aforementioned period, the logarithm of colony-forming units per gram (log_10_ cfu/g) was utilized to calculate the bacterial counts on each plate [47–49].

### 2.11. Assessment of TCC

By using spectrophotometric analysis, the TCC of prawn samples (including the hepatopancreas, shell, and muscle) was quantitatively assessed and expressed in micrograms per gram of dry weight (μg/g). For this analysis, 1 g of specimens after the homogenization process was individually placed into conical centrifuge tubes (Stemcell Technologies, Falcon, Vancouver, Canada). Afterward, 10 mL of dimethyl ketone (KTA, Purity ≥98%, Tehran, Iran) and 2 g of sodium sulfate anhydrous (Na_2_SO_4_; Merck Millipore, EMSURE CAS No. 7757-82-6, USA) were introduced to each sample, which was then thoroughly mixed using a homogenizer for a period of 10 min. The resulting mixtures that were prepared underwent filtration using qualitative filter papers (Whatman, Model G-4, USA) and were subsequently purified by the addition of three aliquots of 10 mL dimethyl ketone, after which centrifugation was performed at a rotational speed of 3500 rpm for a duration of 10 min. The absorbance of the liquid phase was then evaluated employing a spectrophotometer (Biochrom, Model Libra S22-UV/Vis, Cambridge, UK) at a wavelength of 450 nm [24, 50].

### 2.12. Assessment of Body Biochemical Compositions

The biochemical composition of the whole body, encompassing ash content, crude protein, crude lipid, and moisture, was assessed in accordance with the methodologies outlined by the AOAC [30]. Specifically, moisture content was quantified by drying the samples in an oven (Behsan, Model BE55, Tehran, Iran) at 105°C until a constant weight was attained. The determination of crude protein was conducted utilizing the Kjeldahl method (*N* × 6.25) employing a Kjeldahl apparatus (Bakhshi, Model SO-R30, Tehran, Iran), while crude lipid content was determined via the Soxhlet extraction procedure utilizing a Soxhlet extractor (Bakhshi, Model KG6-500, Tehran, Iran). The ash content was measured using a laboratory chamber furnace (Koyo Thermo Systems Co., Nara, Japan) at a temperature of 550°C for a duration of 8 h [32, 48, 51, 52].

### 2.13. Assessment of Amino Acid and Fatty Acid Compositions

The examination of amino acids was performed in accordance with the methodology established by Li et al. [53]. Following the freeze–drying process, the muscle tissue sample underwent hydrolysis using hydrochloric acid 6 mol/L (Sigma–Aldrich, Model Titripur, Missouri, USA). Subsequently, the hydrolyzed combination was subjected to a series of processes including dilution, evaporation, resuspension, and an additional dilution prior to filtration (Merck Millipore, Model Millex-GP Filter Unit, Polyethersulfone pore size 0.22 μm/diameter 33 mm, Massachusetts, USA). The amino acid profiles were then subjected to quantitative analysis utilizing an amino acid analysis system (Hitachi High-Tech Corporation, Model VWR LA8080, Tokyo, Japan).

The fatty acid analysis was performed utilizing a modified version of the methodology established by Chen et al. [54]. Specifically, 0.1 g of the muscle tissue specimen was combined with 3 mL of a methanolic potassium hydroxide solution c(KOH) (Sigma–Aldrich, Model Titripur, Catalog No.109351, Missouri, USA) and subjected to a stable temperature of 72°C in a water bath for a duration of 20 min. Following this, the mixture was allowed to cool at 25°C, after which 3 mL of methanolic hydrogen chloride solution (Sigma–Aldrich, Model LiChropur, CAS No. 7647-01-0, Missouri, USA) was introduced, and the sample was again held at a temperature of 72°C for an additional 20 min. After being cooled at 25°C, 1 mL of n-hexane (Sigma–Aldrich, Model MS SupraSolv, CAS No. 110-54-3, Missouri, USA) was incorporated into the mixture and permitted to remain at this temperature for a duration of 8 h. Subsequently, the resulting supernatant was subjected to centrifugation at a speed of 10,000 rpm for a duration of 2 min. The analysis of the fatty acid compositions was conducted utilizing gas chromatography (Agilent Technologies, Model 7890B GC, California, USA).

### 2.14. Assessment of Growth and Immune-Related Gene Expression

For the purpose of RNA extraction, a sample of 100 mg of hepatopancreas specimens was obtained. Following the homogenization of the samples, 1 mL of the RNA extraction kit (SINACLON, RNX-Plus, Cat. No. EX6101, Tehran, Iran) was utilized in accordance with the manufacturer's guidelines. The integrity of RNA was assessed through electrophoresis (Bio-Rad, Model Sub-Cell GT, California, USA) on an agarose gel (EURx, 1%, Catalog No. E0301-500, Gdańsk, Poland). Additionally, the purity and concentration of the extracted RNA were quantified spectrophotometrically (Thermo Fisher Scientific, Model NanoDrop 2000c, Massachusetts, USA) by measuring the OD260/OD280 ratio. Before cDNA synthesis, the DNase I reaction protocol was used based on the method suggested by the Fermentas kit (Fermentas Life Sciences, Model DNase I, RNase free-EN0521, Massachusetts, USA). Then, cDNA was synthesized with a reverse transcriptase cDNA synthesis kit (SMOBIO, Model ExcelRT series, Hsinchu, Taiwan). Before utilization, the resultant cDNA was preserved at a temperature of −80°C. The gene sequences of *M. nipponense* were obtained from the National Center for Biotechnology Information (NCBI) database (https://www.ncbi.nlm.nih.gov/), while the primer sequences were developed using the NCBI Primer BLAST tool (https://www.ncbi.nlm.nih.gov/tools/primer-blast).  Table 2 presents the synthesized primer sequences. The Ampliqon (RealQ Plus 2x Master Mix Green, High ROX, Odense, Denmark) was employed to assess the expression of immune-related genes in prawns. Quantitative reverse transcription polymerase chain reaction (qRT-PCR) was conducted using an Applied Biosystems device (StepOnePlus, VeriFlex-96 Well, California, USA). The reaction mixture, totaling 25 μL, comprised 12.5 μL of Ampliqon-RealQ Plus 2x Master Mix Green, 2 μL of cDNA template, and 1 μL (10 μM) of both forward and reverse primers. The thermal cycling conditions comprised an initial pre-denaturation step at 95°C for a duration of 30 s, succeeded by a denaturation step at 95°C for 30 s, an annealing phase at 60°C for 30 s, and a total of 40 cycles were conducted. Each specimen was examined three times, and the levels of gene expression were determined utilizing the 2^−*ΔΔ*Ct^ method, with normalization performed against *β*-actin expression [8, 43, 52].

### 2.15. Analysis of Statistical Data

Data analysis was performed utilizing one-way ANOVA through the statistical software (IBM SPSS, Version 22, New York, USA). Additionally, the normality of the data assessment was conducted employing the Kolmogorov–Smirnov test, whereas the homogeneity of variances was determined through the Levene test. Duncan's multiple range test was used to calculate the mean values of the treatments, with a confidence level set at 95%. Eventually, the spreadsheet software (Microsoft Excel 2016, Washington, USA) was used to organize the data into tables, and the textual data was presented in the format of (mean ± standard deviation).

## 3. Results

### 3.1. Water Physicochemical Quality Parameters

The findings regarding water quality indicators are illustrated in Table 3. The results indicate that the treatments containing *β*-carotene administration have a significant influence on any of the measured parameters, as there were no substantial differences observed when compared to the treatment without supplementation (*p* > 0.05).

### 3.2. Growth Metrics

The outcomes regarding the growth metrics investigation of prawns subjected to varying dietary additions of *β*-carotene pigment are summarized in Table 4. The data indicate that an elevation in *β*-carotene levels correlates with enhanced growth indices, FCR, and SR of the prawns, with differences that are statistically significant in comparison to the nonaddition of *β*-carotene pigment group (*p* < 0.05). Conversely, HSI did not exhibit any notable variation among the experimental treatments (*p* > 0.05).

### 3.3. Hemolymph Biochemical Indicators

The findings derived from the assessments of biochemical indices in the studied prawn's hemolymph subjected to diets with varying levels of *β*-carotene are shown in Table 5. The data indicate that an increase in dietary *β*-carotene levels is linked to a notable reduction in the concentrations of triglycerides, uric acid, glucose, urea, and creatinine in contrast to the without *β*-carotene group (*p* < 0.05). Notably, the control treatment exhibited the lowest levels of HDL and LDL. Furthermore, there were no statistically notable differences in phosphorus, calcium, and cholesterol indices across the *β*-carotene supplementation groups (*p* > 0.05).

### 3.4. Immunological Parameters

Table 6 shows the results obtained from the examination of immunological parameters in the prawns. Notably, the levels of ALB and TP exhibited a significant increase in prawns subjected to *β*-carotene treatments. Conversely, the CORT levels in prawns receiving *β*-carotene demonstrated a significant reduction in comparison to the no *β*-carotene treatment (*p* < 0.05). Additionally, the parameters related to cellular immunity (such as SGC, THC, HC, and GC) of prawns treated with varying concentrations of *β*-carotene demonstrated a noteworthy enhancement relative to the nonaddition of *β*-carotene pigment group (*p* < 0.05). The activity of PO and LYZ in the *β*-carotene supplementation groups was elevated compared to the without *β*-carotene group (*p* < 0.05). Furthermore, the levels of AST, LDH, and ALT exhibited an obvious decrease in comparison to the no *β*-carotene addition group (*p* < 0.05) and the levels of AKP exhibited no significant changes in response to the differing concentrations of *β*-carotene (*p* > 0.05).

### 3.5. Antioxidant Activities

The findings regarding antioxidant parameters are summarized in Table 7. A remarkable increase in T-AOC of *β*-carotene-treated groups was noted in comparison to the group without *β*-carotene pigment. Conversely, the levels of MDA, SOD, and CAT exhibited a statistically notable decrease with regard to *β*-carotene treatments (*p* < 0.05). It is noteworthy that the activity of GPx remained unaffected by varying concentrations of *β*-carotene (*p* > 0.05).

### 3.6. Digestive Enzyme Activity

Table 8 presents the findings from the determination of digestive enzyme activity. The results indicate that varying levels of *β*-carotene significantly influenced the activity of digestive enzymes, with a notable increase in the digestive enzyme activities, including amylase, protease, and lipase in prawns as dietary *β*-carotene levels increased (*p* < 0.05). The treatment group that administered 200 mg/kg of *β*-carotene pigment exhibited the highest enzyme activities; on the other hand, the treatment with no *β*-carotene supplementation demonstrated the lowest enzyme activities (*p* < 0.05).

### 3.7. Intestinal Microflora

Results obtained from the analysis of intestinal microflora are illustrated in Table 9. The findings indicate that TBC exhibited a statistically significant difference across various treatments, demonstrating a notable decrease in comparison to the nonadministered *β*-carotene pigment group (*p* < 0.05). Conversely, lactic LAB illustrated no statistically noteworthy variation in response to differing dietary levels of *β*-carotene (*p* > 0.05).

### 3.8. TCC

The findings regarding the TCC in different body parts of prawns are summarized in Table 10. The treatment receiving 200 mg/kg of dietary *β*-carotene exhibited the highest levels of the TCC in the shell, hepatopancreas, and muscle tissues, while on the contrary the treatment that did not receive *β*-carotene supplementation demonstrated the lowest levels (*p* < 0.05).

### 3.9. Body Compositions

Table 11 shows findings obtained from the carcass analysis of the prawns. The data indicate that the moisture content in the *β*-carotene treatments exhibited a significant reduction, while the crude lipid and crude protein levels demonstrated a notable increase when compared to the group that did not receive *β*-carotene supplementation (*p* < 0.05). Conversely, the assessment of crude ash revealed no remarkable differences among the experimental treatments (*p* > 0.05).

### 3.10. Amino Acid and Fatty Acid Profiles

Table 12 presents the findings from the evaluations of the whole-body amino acid profiles and results regarding the whole-body fatty acid compositions are illustrated in Table 13. The data indicate that the profiles of amino acids and fatty acids in prawns that received *β*-carotene pigment showed significant improvements. An increase in dietary *β*-carotene led to a substantial rise in polyunsaturated fatty acids (PUFAs), monounsaturated fatty acids (MUFAs), and essential amino acids (EAAs), with these changes being statistically significant in comparison to the control group (*p* < 0.05). However, an elevation in *β*-carotene concentrations within the experimental diets was associated with a reduction in the levels of certain non-EAAs (NEAAs) and saturated fatty acids (SFAs) in comparison to the nonsupplemented *β*-carotene treatment (*p* < 0.05).

### 3.11. Expression of Genes Related to Growth and Immunity

**Figure 1:**
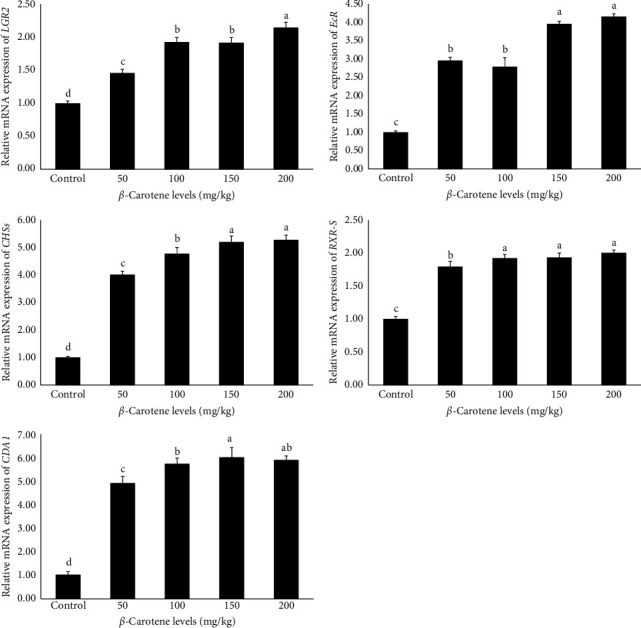
The role of dietary *β*-carotene supplementation in the oriental river prawn: Impacts on growth-related gene expression following an 8-week feeding trial. Values are presented as mean ± standard deviation (M ± SD, *n* = 3). A significant difference between the treatments is indicated by means in bars with different superscript letters (*p* < 0.05).

**Figure 2:**
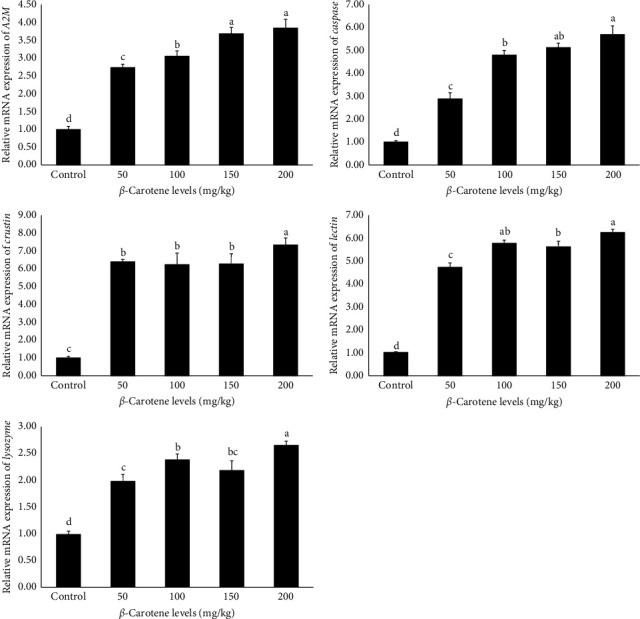
The role of dietary *β*-carotene supplementation in the oriental river prawn: Impacts on immune-related gene expression following an 8-week feeding trial. Values are presented as mean ± standard deviation (M ± SD, *n* = 3). A significant difference between the treatments is indicated by means in bars with different superscript letters (*p* < 0.05).

The findings derived from the analysis of the relative mRNA expression of genes related to growth and immunity are presented in Figures 1 and 2, respectively. The data indicate that the relative expression level of all genes, including *retinoid X receptor* (*RXR-S*), *lectin*, *ecdysteroid receptor* (*EcR*), *LYZ*, *caspase*,*leucine-rich repeat-containing G-protein-coupled receptor 2* (*LGR2*), *alpha-2-macroglobulin* (*A2M*), *cytidine deaminase 1* (*CDA1*), *crustin*, and *chitin synthase* (*CHSs*), were significantly influenced by different levels of *β*-carotene supplementation (*p* < 0.05). Specifically, the results demonstrated that an increase in dietary *β*-carotene levels corresponded with a notable rise in the relative mRNA expression of the aforementioned genes among the experimental treatments. Notably, the treatments containing 150 and 200 mg/kg of *β*-carotene exhibited the highest relative expressions, which was notably different from the other treatments. In comparison, the control treatment exhibited the lowest relative expression levels of genes associated with growth and immune functions (*p* < 0.05).

## 4. Discussion

### 4.1. Growth Performance

The results of our work express that the incorporation of dietary *β*-carotene leads to a significant enhancement in growth metrics in comparison to the control. These findings align with previous research examining the impact of carotenoid pigments on the aforementioned indices of different crustacean species, including the investigations conducted by Fawzy et al. [24] and Shen et al. [55] on white shrimp (*L. vannamei*), also Deng et al. [56] on swimming crab (*Portunus trituberculatus*), Zhang et al. [57] on red swamp crayfish (*P. clarkii*), Wang et al. [58] on giant tiger shrimp (*Penaeus monodon*), and Wang et al. [44] on kuruma shrimp (*Marsupenaeus japonicus*). A comparative examination of findings from analogous experiments alongside the present research indicates that increased dietary intake of carotenoids, particularly *β*-carotene, positively influences growth indices. This improvement can be attributed to the significant role that these carotenoid pigments serve as mediators in metabolic processes, thereby, enhancing food efficiency through the acceleration of digestion [59, 60]. Furthermore, carotenoids contribute to a reduction in energy expenditure by decreasing the duration of the molting intervals in crustaceans and inhibiting NADPH activity, which in turn facilitates optimal growth in these aquatics [59–62].

### 4.2. Hematological Parameters

In terms of hemolymph biochemical indices measured in our study, indicate that an increase in dietary *β*-carotene supplementation correlates with a significant reduction in the values of most aforementioned parameters. Conversely, HDL and LDL levels showed a significant increase relative to the control group. Additionally, calcium, phosphorus, and cholesterol levels remained unchanged. These findings align with the feeding trial conducted by Fawzy et al. [6] and Liu et al. [60] on white shrimp (*L. vannamei*), also Wang et al. [58] on giant tiger shrimp (*P. monodon*). Given that glucose serves as a short-term stress index in aquatic animals, the observed reduction in glucose levels within the *β*-carotene-fed groups suggests a potentially advantageous function of carotenoids in alleviating physiological stress [1, 63, 64]. Additionally, in the groups containing *β*-carotene, triglyceride levels were also significantly reduced relative to the control treatment. This finding supports the hypothesis that carotenoids confer protective effects as opposed to oxidative stress, leading to a decrease in triglyceride accumulation through the esterification process, which involves the interaction of carotenoid pigments with fatty acids [65].

### 4.3. Immunological Responses

Our study findings indicate that, dietary *β*-carotene addition significantly improved immunological responses in comparison to the nonsupplemented treatment. The results of current work are compatible with previous research regarding the impact of astaxanthin on the abovementioned parameters, as documented by the works of Eldessouki et al. [66], Chuchird, Rorkwiree, and Rairat [67], and Flores et al. [68] on *L. vannamei*, as other studies conducted by Cheng and Wu [25] on *P. clarkii*, Weilong et al. [45] and Wang et al. [44] on *M. japonicus*. The findings derived from the aforementioned studies underscore the crucial function of carotenoid pigments in enhancing the nonspecific immunity and augmenting resistance to environmental pathogens, including bacteria, as well as improving tolerance to environmental stressors. In general, an elevation of THC in crustaceans is regarded as an indicator of disease resistance [25, 66, 67, 69]. In the present research, it was observed that the levels of LYZ and PO in groups supplemented with *β*-carotene were notably elevated compared to treatment without such supplementation. The aforementioned results are consistent with the study findings presented by Cheng and Wu [25], which examined the impact of astaxanthin on the activity of LYZ enzyme in *P. clarkii*. Commonly, LYZ and PO levels are recognized as critical markers of innate immune system responses in crustacean species, playing a significant role in bactericidal activity, particularly against gram-positive bacteria. The activity of these enzymes serves as a reliable indicator of the immune competence and total health conditions of these aquatics [25, 66, 69, 70]. In our work, we observed that increasing *β*-carotene supplementation levels resulted in a significant enhancement of immune-related genes. This result aligns with the feeding trials administered by Zhang et al. [43] and Niu et al. [8] for investigation impacts of astaxanthin pigment on the expression of genes related to growth and immunity of *L. vannamei* and *P. monodon*, respectively. Researchers have demonstrated that carotenoids enhance the expression of toll-like receptors (TLRs), which play a crucial role in activating the innate immune system of crustaceans. These receptors function by recognizing specific patterns and transmitting signals to pattern recognition receptors (PRRs). This series of events, initiated by the stimulation of the immune system in crustaceans, subsequently leads to the expression of various immune-related genes [8, 43, 52]. Nonetheless, the impact of carotenoids on the mechanisms associated with the expression of genes involved in growth and immunity is not yet fully understood and necessitates additional investigation in this area of study. A comparative analysis of our work with similar studies suggests that the observed elevation in hemolymph enzymatic activity levels within the examined specimens underscores the notable influence of carotenoids, particularly *β*-carotene, on the innate immune responses of the prawn. The findings indicate that the LDH, AST, and ALT enzyme activities exhibited an obvious reduction in relation to the control group, whereas AKP remained unaffected by varying concentrations of *β*-carotene. These outcomes align with the findings reported by Chen et al. [54] and Wang et al. [58] on *P. monodon* and also Zhang et al. [57] on *P. clarkii*. The enzymes AST and ALT act as critical transporters of amino acids and are integral to protein metabolic pathways; thus, fluctuations in these enzyme values can reflect the physiological condition of hepatopancreatic cells. In general, previously stated enzymes are present at low concentrations; however, in instances of severe cellular damage, elevated levels are released into the hemolymph [25, 55, 71]. The observed similarities between the findings of our research and the abovementioned investigation regarding the notable decrease in these enzyme levels following *β*-carotene treatment underscore the important function of this carotenoid pigment, akin to certain others, in enhancing the condition of hepatopancreas in the studied prawns.

### 4.4. Antioxidant Activities

Recent research findings showed that elevated dietary levels of *β*-carotene are correlated with a substantial enhancement in T-AOC in comparison to the group with no *β*-carotene addition. This observation is connected to previous studies examining the influence of varying levels of carotenoids on juvenile Chinese mitten crab (*E. sinensis*), juvenile *L. vannamei*, and *P. monodon*, including works carried out by Jiang et al. [72], Fang et al. [62], Niu et al. [8], Chien, Pan, and Hunter [73], and Pan, Chien, and Hunter [74], respectively. These studies similarly concluded that an elevation in levels of dietary astaxanthin pigment led to a notable enhancement of T-AOC relative to the nonastaxanthin group. Typically, T-AOC serves as a comprehensive measure of both enzymatic and nonenzymatic antioxidant activities within the biological system, reflecting the organism's response to a range of beneficial or detrimental factors. The variations in the concentrations of the mentioned antioxidants may exhibit either an upward or downward trend. This parameter is crucial for evaluating the equilibrium between oxidant and antioxidant elements; an increase in T-AOC is indicative of enhanced tolerance to oxidative stress in aquatics [56, 62, 69, 75]. A comparison of our results with those from analogous research that demonstrates an increase in T-AOC in subjects receiving carotenoid pigment treatments suggests the notable antioxidant efficacy of these pigments. Present work findings indicated that an elevation in *β*-carotene supplementation was linked with a significant reduction in the MDA, CAT, and SOD levels. These results agree with the study carried out by Zhang et al. [43], which examined these indices in *L. vannamei* and reported a notable decrease in SOD and CAT enzyme activities when subjected to elevated dietary astaxanthin levels contrasted to the control group. Additionally, investigations by Chen et al. [54], Wang et al. [58], and Niu et al. [8] on *P. monodon*, also Cheng and Wu [25] on *P. clarkii* and Han et al. [42] on *P. trituberculatus* also demonstrated a remarkable reduction in MDA levels in astaxanthin-fed experimental groups relative to the nonsupplemented treatment, corroborating the findings of the current research. SOD and CAT are critical components of the primary antioxidant protection mechanism against free radicals, which mitigates detrimental effects induced by these oxidant compounds. These enzymes are extremely vital for inhibiting the formation of stated radicals during oxidative processes and are essential for the protection of various cellular membrane structures [76–78]. The results of our work, along with those previously referenced, indicate that the observed reduction in SOD levels parallels the function of carotenoids, like *β*-carotene, in the scavenging of superoxide anion radicals (O_2_^·−^) according to their oxidation–reduction capabilities. This suggests that diminished levels of SOD are employed to neutralize and eliminate earlier-mentioned radicals [77, 79, 80]. Furthermore, the notable reduction in CAT levels observed in subjects receiving *β*-carotene implies a reduced necessity for this enzyme. This phenomenon can be ascribed to the decreased generation of hydrogen peroxide (H_2_O_2_) metabolites, which is linked to the reduced activity of SOD throughout the mitigation process of free radicals facilitated by the carotenoid pigment [80–82]. As previously noted, an increase in additional *β*-carotene levels is linked to a substantial decrease in MDA levels, substantiating results from numerous studies. These outcomes suggest that carotenoids play a crucial role in inhibiting lipid peroxidation (self-propagating chain reaction) that is driven by free radicals. Consequently, the inclusion of carotenoids, such as *β*-carotene, in dietary regimens diminishes lipid peroxidation levels and offers protective effects against free radical-induced damage [82, 83].

### 4.5. Digestive Enzyme Activity

In our study, we observed that increasing *β*-carotene supplementation levels led to a marked enhancement in the activity of digestive enzymes in comparison to the control group. This result aligns with the feeding trial administered by Fawzy et al. [24] and Wang et al. [44], which examined the effects of the carotenoid supplementation on the aforementioned enzyme activities of *L. vannamei* and *M. japonicus*, respectively. The activity of these enzymes is recognized as a crucial indicator of nutritional status and growth modulation in aquatics, thereby, facilitating the preparation of desirable diets for various crustaceans [6, 24, 44, 45]. Carotenoid pigments sustain a low pH in the intestinal environment, thereby, fostering the growth of advantageous bacteria such as *Bacillus* that synthesize digestive enzymes. Carotenoids amplify the activity of these enzymes, thereby, enhancing digestion, absorption, and overall nutritional efficacy through the modulation of bacterial populations [24, 84, 85]. The similarities in findings across different research underscore the important role that carotenoids play in improving nutritional processes in aquatic organisms.

### 4.6. Intestinal Microflora

The current investigation into intestinal microflora revealed a notable variation in TBC among the experimental groups. Previously mentioned outcomes are consistent with the findings reported by Chuchird, Rorkwiree, and Rairat [67], which examined the impact of varying levels of astaxanthin on *L. vannamei*. Carotenoid pigments are recognized for their role in modulating intestinal microbial composition by sustaining an acidic milieu that deters pathogenic organisms while favoring beneficial species. Specifically, carotenoids are effective in diminishing pathogenic strains and augmenting populations of *Bacillus* [24, 58, 67]. The observed decrease in TBC with *β*-carotene administration reinforces these observations, indicating that it enhances the intestinal microbiota by promoting competitive dynamics and preserving an acidic pH conducive to a reduction in the population of pathogenic bacteria.

### 4.7. Body Composition Profiles

In our study, both crude lipid and crude protein levels exhibited a significant increase in response to elevated dietary *β*-carotene. The findings derived from the body-approximate analysis indicated a positive correlation between higher *β*-carotene supplementation levels in dietary interventions and enhancements in body composition. This investigation supports the conclusions drawn by Jiang et al. [23] on *E. sinensis*, Göçer et al. [86] regarding the grooved tiger shrimp (*Penaeus semisulcatus*), as well as those of Wang et al. [58] and Niu et al. [8] in relation to *P. monodon*. The substantial influence of carotenoids on proteins and lipids metabolism provides a rationale for the pronounced effects of the mentioned pigment on biochemical body compositions within the experimental prawn [23, 58, 86]. Additionally, current research demonstrated that an increase in dietary *β*-carotene corresponded with a notable rise in the TCC in different body tissues of the examined prawn. Our work results are similar to the research conducted by Jiang et al. [23] on *E. sinensis*, Fawzy et al. [24] on *L. vannamei*, and Wade et al. [87] on *P. monodon*. Recent research findings indicate that an increase in dietary *β*-carotene is associated with a significant elevation in EAA, MUFA, and PUFA. Conversely, there was a notable decrease in SFA in the groups that received *β*-carotene supplementation. Our results align with research conducted by Chen et al. [54] on *P. monodon*. The beneficial role of carotenoids in augmenting levels of EAA and PUFA may be attributed to their antioxidant characteristics, which protect these elements from oxidative deterioration. Additionally, the observed decrease in SFA associated with carotenoid pigments may result from the activation of desaturation and elongation pathways, thereby, contributing to the observed increase in PUFA levels. Nevertheless, the precise mechanisms by which carotenoid pigments impact amino acid and fatty acid metabolism require further investigation [54, 88, 89].

## 5. Conclusion

Our study revealed that high levels of *β*-carotene supplementation positively influenced the growth indices, biochemical indicators, immune, and metabolic responses of the oriental river prawn. This underscores the critical role of *β*-carotene as a crucial carotenoid in promoting growth metrics, supporting immune system functionality, and digestibility improvement. Overall, based on the observed results, it is recommended to incorporate dietary 200 mg/kg of *β*-carotene supplementation to optimize the aforementioned evaluations in this species.

## Figures and Tables

**Table 1:** Feed ingredients, formulation, and proximate composition analysis (on a dry matter basis) of the experimental diets utilized in the present study.

	*β*-Carotene (mg/kg)				
	Control	50	100	150	200
Ingredients (g/kg)					
Fish meal^a^	300.00	300.00	300.00	300.00	300.00
Soy meal	300.00	300.00	300.00	300.00	300.00
Wheat meal	70.00	70.00	70.00	70.00	70.00
Corn meal	70.00	70.00	70.00	70.00	70.00
Casein^b^	160.00	160.00	160.00	160.00	160.00
Vitamin premix^c^	20.00	20.00	20.00	20.00	20.00
Mineral premix^d^	20.00	20.00	20.00	20.00	20.00
Cholesterol^e^	2.00	2.00	2.00	2.00	2.00
Vitamin C^f^	1.00	1.00	1.00	1.00	1.00
Dicalcium phosphate^g^	5.00	5.00	5.00	5.00	5.00
Filler (CMC) premix^h^	52.00	51.95	51.90	51.85	51.80
Carotenoid pigment (*β*-carotene)^i^	0	0.05	0.10	0.15	0.20
Proximate analysis (% dry matter)					
Moisture	9.59	9.36	9.28	9.53	9.72
Crude protein	44.81	44.67	44.84	44.94	45.06
Crude lipid	5.23	4.48	4.63	4.70	4.58
Crude fiber	2.77	2.79	2.80	2.91	2.83
Ash	14.70	14.85	14.43	14.38	14.29
Nitrogen-free extract	22.90	23.85	24.02	23.54	23.52
Gross energy (kJ/g)^j^	18.48	18.12	18.37	18.41	18.23
Total carotenoids (mg/kg)	5.26	52.64	109.11	148.94	196.89

^a^Arad Podr (Tehran, Tehran, Iran).

^b^Quelab Laboratories Inc. (CAS No. 9000-71-9, Montréal, Canada).

^c^Aras (Aquavit-I, Fars, Iran)—Each 1000 g Vitamin premix contained: Vitamin A (retinol) 1,200,000 IU, Vitamin B_1_ (thiamin) 2500 mg, Vitamin B_2_ (riboflavin) 4000 mg, Vitamin B_3_ (niacin) 35,000 mg, Vitamin B_5_ (pantothenic acid) 10,000 mg, Vitamin B_6_ (pyridoxine) 2500 mg, Vitamin B_7_ (biotin) 150 mg, Vitamin B_9_ (folate) 1000 mg, Vitamin B_12_ (cobalamin) 8 mg, Vitamin C (ascorbic acid) 30,000 mg, Vitamin D_3_ (cholecalciferol) 400.000 IU, and Vitamin K_3_ (menadione) 800 mg.

^d^Science Laboratories (Model SL-0.5, Qazvin, Iran)—Each 1000 g Mineral premix contained: cobalt (Co) 100 mg, copper (Cu) 600 mg, choline chloride 6000 mg, iodine (I) 600 mg, iron (Fe) 6000 mg, manganese (Mn) 5000 mg, selenium (Se) 20 mg, and zinc (Zn) 10,000 mg.

^e^Merck Group (Sigma–Aldrich, CAS No. 57-88-5, St. Louis, USA).

^f^Aras (Aquavit-C, Fars, Iran)—Each 500 g Vitamin C premix contained: Stay-C 35%.

^g^Aras (CAS No. 7757-93-9, Tehran, Iran).

^h^Kimia-Acid Laboratories Inc. (Tehran, Tehran, Iran).

^i^DSM Nutritional Products AG (10% CWS/S, Basel, Switzerland).

^j^The calculation is based on values of 16.7, 37.6, and 16.7 kJ/g for protein, lipid, and carbohydrate, respectively.

**Table 2:** Nucleotide sequences and sources of primers used for quantitative reverse transcription polymerase chain reaction (qRT-PCR) in the present study.

Gene name	Primer nucleotide sequence (5′ to 3′)	Product length (bp)	GenBank accession No.
*Leucine-rich repeat-containing G-protein-coupled receptor 2* (*LGR2*)	F: TCCTTCCACTGGTCAGCATT R: CAGTGGTAGGCGTAGGAGAG	184	MT585155.1

*Ecdysteroid receptor* (*EcR*)	F: AGAACCCTCGGCAATCTCAA R: CCTTCCTCCTTCCTTCCTCG	178	MH459143.1

*Chitin synthase* (*CHSs*)	F: GCATTGAGTGGCAGCTTCTT R: GGCTATCTCTCGTGTACCCC	168	KP710198.1

*Retinoid X receptor* (*RXR-S*)	F: CCTCTCCCAGTGTGTCCAAT R: ACCTTTGCAACCCTCACAAC	197	KC460324.1

*Cytidine deaminase 1* (*CDA1*)	F: AAGAACGCCTTCCTGTACGA R: ATCCGGGCAAGTCTTCTTCA	205	MF360010.1

*Alpha-2-macroglobulin* (*A2M*)	F: CGGCAGTAATGAACGTCCAG R: GCGAGGCTGAGAGGGATATT	160	MK439847.1

*Caspase*	F: TGCATGACAAGCCCAGACTA R: TGACCAAACTCCTGCAATGC	217	KU942381.1

*Crustin*	F: ACCCGTTACCAGCTTCTTCA R: AAGGGAAACGCTGCTTTACG	241	OM032597.1

*Lectin*	F: GCTGACGGACCAAGCCTATA R: ATTCCCGTTATGAGGCGTGA	175	PP516428.1

*Lysozyme* (*LYZ*)	F: CGACACCGAACGCTACAAGG R: GGAACCACGAGACCAGCAC	118	AY257550.2

*β-actin*	F: GTGCCCATCTACGAGGGTTA R: CGTCAGGGAGCTCGTAAGAC	247	KY780298.1

**Table 3:** The role of dietary *β*-carotene supplementation in the oriental river prawn: Impacts on water quality indicators following an 8-week feeding trial.

Parameters	*β*-Carotene (mg/kg)					One-way ANOVA		
	Control	50	100	150	200	*F*	d.f.	*p*-Value
Temperature (°C)	24.88 ± 0.25	25.35 ± 0.79	24.88 ± 0.15	25.12 ± 0.34	25.87 ± 1.01	0.331	4	0.851
pH	6.84 ± 0.07	6.91 ± 0.19	6.88 ± 0.29	7.04 ± 0.25	7.06 ± 0.13	0.678	4	0.623
Dissolved oxygen (mg/L)	6.91 ± 0.23	6.97 ± 0.11	6.94 ± 0.45	6.98 ± 0.41	7.02 ± 0.30	1.884	4	0.190
Ammonium (mg/L)	0.83 ± 0.03	0.79 ± 0.04	0.83 ± 0.07	0.78 ± 0.05	0.80 ± 0.02	0.928	4	0.485
Nitrite (mg/L)	0.13 ± 0.01	0.11 ± 0.01	0.12 ± 0.03	0.11 ± 0.02	0.11 ± 0.02	0.881	4	0.534
Nitrate (mg/L)	0.18 ± 0.01	0.18 ± 0.02	0.19 ± 0.01	0.18 ± 0.02	0.17 ± 0.01	1.045	4	0.431
Phosphate (mg/L)	0.02 ± 0.02	0.02 ± 0.01	0.02 ± 0.02	0.02 ± 0.01	0.01 ± 0.02	0.929	4	0.485
Total hardness (mg/L)	135.73 ± 2.85	131.84 ± 1.70	132.26 ± 2.40	134.70 ± 2.62	132.27 ± 2.68	1.579	4	0.254

*Note:* Values are presented as mean ± standard deviation (M ± SD, *n* = 3).

**Table 4:** The role of dietary *β*-carotene supplementation in the oriental river prawn: Impacts on growth performance following an 8-week feeding trial.

Parameters	*β*-Carotene (mg/kg)					One- way ANOVA		
	Control	50	100	150	200	*F*	d.f.	*p*-Value
Final body weight (g)	4.87 ± 0.12^d^	5.45 ± 0.22^c^	6.14 ± 0.35^b^	6.73 ± 0.31^a^	6.94 ± 0.47^a^	18.403	4	0.001
Weight gain (WG; g)	3.51 ± 0.17^d^	4.08 ± 0.25^c^	4.78 ± 0.41^b^	5.36 ± 0.39^a^	5.58 ± 0.62^a^	11.019	4	0.001
Percent WG (%)	256.20 ± 19.41^d^	297.81 ± 24.73^c^	348.91 ± 24.05^b^	391.24 ± 27.12^a^	407.30 ± 25.68^a^	11.021	4	0.001
Specific growth rate (SGR; %/day)	2.26 ± 0.08^b^	2.47 ± 0.14^ab^	2.68 ± 0.06^ab^	2.84 ± 0.10^a^	2.90 ± 0.12^a^	5.816	4	0.014
Feed conversion ratio (FCR)	1.94 ± 0.03^a^	1.85 ± 0.02^b^	1.68 ± 0.05^c^	1.59 ± 0.03^d^	1.41 ± 0.06^e^	364.710	4	0.001
Hepatosomatic index (HSI; %)	4.71 ± 0.16	4.88 ± 0.35	4.75 ± 0.40	5.03 ± 0.52	4.98 ± 0.32	0.522	4	0.746
Survival rate (SR; %)	91.11 ± 7.70^b^	95.55 ± 3.85^ab^	100.00 ± 0.00^a^	100.00 ± 0.00^a^	100.00 ± 0.00^a^	3.127	4	0.044

*Note:* Values are presented as mean ± standard deviation (M ± SD, *n* = 3). A significant difference between the treatments is indicated by means in the same row with different superscript letters (*p* < 0.05).

**Table 5:** The role of dietary *β*-carotene supplementation in the oriental river prawn: Impacts on hemolymph biochemical indicators following an 8-week feeding trial.

Parameters	*β*-Carotene (mg/kg)					One- way ANOVA		
	Control	50	100	150	200	*F*	d.f.	*p*-Value
Urea (mg/dL)	12.25 ± 0.30^a^	11.37 ± 0.19^b^	11.40 ± 0.22^b^	11.14 ± 0.26^c^	11.05 ± 0.17^c^	29.934	4	0.001
Uric acid (mg/dL)	1.47 ± 0.11^a^	1.43 ± 0.15^b^	1.36 ± 0.20^ab^	1.33 ± 0.09^ab^	1.28 ± 0.15^c^	16.525	4	0.001
Glucose (mg/dL)	38.37 ± 0.35^a^	36.44 ± 0.42^b^	35.83 ± 0.26^c^	36.47 ± 0.21^b^	35.03 ± 0.33^d^	103.138	4	0.001
Creatinine (mg/dL)	0.21 ± 0.02^a^	0.19 ± 0.01^b^	0.17 ± 0.01^bc^	0.17 ± 0.02^bc^	0.14 ± 0.01^c^	7.962	4	0.006
Calcium (mg/dL)	68.91 ± 1.79	69.45 ± 2.39	68.49 ± 1.52	69.96 ± 1.38	68.35 ± 1.66	0.417	4	0.794
Phosphorus (mg/dL)	13.06 ± 0.59	13.21 ± 0.69	13.02 ± 0.35	13.18 ± 0.33	13.10 ± 0.24	0.082	4	0.985
Cholesterol (mg/dL)	46.89 ± 0.63	47.01 ± 0.14	46.77 ± 0.88	46.68 ± 0.15	46.47 ± 0.55	0.412	4	0.801
Triglycerides (mg/dL)	75.08 ± 0.51^a^	71.22 ± 0.30^b^	71.10 ± 0.24^b^	69.33 ± 0.85^c^	66.05 ± 0.42^d^	129.016	4	0.001
High-density lipoprotein (HDL; mg/dL)	10.17 ± 0.39^d^	11.28 ± 0.32^c^	12.36 ± 0.43^b^	11.90 ± 0.30^bc^	13.22 ± 0.59^a^	21.602	4	0.001
Low-density lipoprotein (LDL; mg/dL)	4.32 ± 0.30^d^	4.91 ± 0.14^c^	5.60 ± 0.19^b^	6.16 ± 0.18^a^	6.27 ± 0.21^a^	50.981	4	0.001

*Note:* Values are presented as mean ± standard deviation (M ± SD, *n* = 3). A significant difference between the treatments is indicated by means in the same row with different superscript letters (*p* < 0.05).

**Table 6:** The role of dietary *β*-carotene supplementation in the oriental river prawn: Impacts on hemato-immune indices following an 8-week feeding trial.

Parameters	*β*-Carotene (mg/kg)					One- way ANOVA		
	Control	50	100	150	200	*F*	d.f.	*p*-Value
Albumin (ALB; g/dL)	1.16 ± 0.02^d^	1.30 ± 0.08^c^	1.39 ± 0.05^b^	1.45 ± 0.06^a^	1.47 ± 0.04^a^	64.793	4	0.001
Total protein (TP; g/dL)	3.61 ± 0.07^e^	5.17 ± 0.14^c^	4.75 ± 0.26^d^	6.02 ± 0.09^b^	6.25 ± 0.07^a^	207.486	4	0.001
Cortisol (CORT; ng/mL)	14.49 ± 0.19^a^	10.77 ± 0.22^d^	12.30 ± 0.28^b^	11.26 ± 0.32^c^	10.43 ± 0.82^d^	98.633	4	0.001
Lysozyme (LYZ; U/min/mL)	10.41 ± 0.28^d^	12.35 ± 0.17^c^	15.01 ± 0.33^b^	15.39 ± 0.31^ab^	15.52 ± 0.40^a^	261.366	4	0.001
Phenoloxidase (PO; U/min/mg protein)	0.47 ± 0.03^d^	0.51 ± 0.02^cd^	0.55 ± 0.02^bc^	0.59 ± 0.01^ab^	0.62 ± 0.05^a^	18.192	4	0.001
Total hemocyte count (THC; ×10^5^ cells/mL)	88.29 ± 1.68^d^	104.77 ± 2.84^c^	121.36 ± 1.59^b^	134.52 ± 2.73^a^	133.26 ± 1.18^a^	262.141	4	0.001
Granular cells (GCs; ×10^5^ cells/mL)	9.07 ± 1.40^c^	14.65 ± 1.71^b^	14.91 ± 2.56^b^	19.79 ± 2.35^a^	18.97 ± 1.36^a^	47.872	4	0.001
Semi-GCs (SGCs; ×10^5^ cells/mL)	35.42 ± 1.19^d^	40.83 ± 2.56^c^	52.74 ± 1.30^b^	56.48 ± 1.83^a^	54.81 ± 1.76^ab^	84.230	4	0.001
Hyaline cells (HCs; ×10^5^ cells/mL)	45.38 ± 1.80^d^	51.72 ± 3.33^c^	54.12 ± 2.64^b^	58.33 ± 2.62^a^	57.97 ± 2.54^a^	98.412	4	0.001
Alanine aminotransferase (ALT; U/L)	23.96 ± 2.13^a^	20.30 ± 1.19^ab^	19.80 ± 0.61^b^	16.39 ± 1.05^c^	14.26 ± 0.58^d^	33.119	4	0.001
Aspartate aminotransferase (AST; U/L)	72.47 ± 1.02^a^	69.12 ± 1.03^b^	68.36 ± 0.70^b^	65.42 ± 1.39^c^	64.35 ± 0.82^c^	37.926	4	0.001
Alkaline phosphatase (AKP; U/L)	155.09 ± 1.98	156.86 ± 1.78	155.92 ± 1.12	156.81 ± 1.94	159.63 ± 2.05	3.755	4	0.146
Lactate dehydrogenase (LDH; U/L)	673.89 ± 5.92^a^	672.11 ± 6.02^a^	663.22 ± 2.57^b^	662.05 ± 3.68^b^	655.73 ± 3.77^c^	8.312	4	0.004

*Note:* Values are presented as mean ± standard deviation (M ± SD, *n* = 3). A significant difference between the treatments is indicated by means in the same row with different superscript letters (*p* < 0.05).

**Table 7:** The role of dietary *β*-carotene supplementation in the oriental river prawn: Impacts on hepatopancreatic antioxidant activities following an 8-week feeding trial.

Parameters	*β*-Carotene (mg/kg)					One- way ANOVA		
	Control	50	100	150	200	*F*	d.f.	*p*-Value
Total antioxidant capacity (T-AOC; U/mg protein)	2.42 ± 0.26^d^	2.62 ± 0.17^c^	3.43 ± 0.14^b^	3.56 ± 0.17^ab^	3.73 ± 0.31^a^	33.882	4	0.001
Superoxide dismutase (SOD; U/mg protein)	7.19 ± 0.41^a^	6.58 ± 0.49^b^	6.10 ± 0.42^bc^	5.89 ± 0.26^c^	5.12 ± 0.47^c^	17.915	4	0.001
Glutathione peroxidase (GPx; U/mg protein)	29.41 ± 1.51	29.13 ± 2.09	26.34 ± 1.62	26.07 ± 1.83	27.52 ± 1.97	2.465	4	0.119
Catalase (CAT; U/mg protein)	14.77 ± 1.09^a^	10.98 ± 1.01^b^	9.25 ± 0.66^c^	10.14 ± 0.59^bc^	9.02 ± 0.41^c^	43.705	4	0.001
Malondialdehyde (MDA; nmol/mg protein)	8.28 ± 0.17^a^	6.49 ± 0.50^b^	6.12 ± 0.61^c^	5.26 ± 0.42^d^	5.17 ± 0.56^d^	20.146	4	0.001

*Note:* Values are presented as mean ± standard deviation (M ± SD, *n* = 3). A significant difference between the treatments is indicated by means in the same row with different superscript letters (*p* < 0.05).

**Table 8:** The role of dietary *β*-carotene supplementation in the oriental river prawn: Impacts on digestive enzyme activities following an 8-week feeding trial.

Parameters	*β*-Carotene (mg/kg)					One- way ANOVA		
	Control	50	100	150	200	*F*	d.f.	*p*-Value
Protease (U/mg protein)	1.31 ± 0.03^d^	1.44 ± 0.02^c^	1.51 ± 0.04^bc^	1.56 ± 0.01^b^	1.63 ± 0.07^a^	34.657	4	0.001
Lipase (U/mg protein)	0.68 ± 0.04^d^	0.77 ± 0.01^c^	0.82 ± 0.03^bc^	0.87 ± 0.03^b^	0.94 ± 0.02^a^	29.518	4	0.001
Amylase (U/mg protein)	1.73 ± 0.09^d^	2.15 ± 0.12^c^	2.39 ± 0.07^b^	2.62 ± 0.08^a^	2.51 ± 0.07^ab^	42.913	4	0.001

*Note:* Values are presented as mean ± standard deviation (M ± SD, *n* = 3). A significant difference between the treatments is indicated by means in the same row with different superscript letters (*p* < 0.05).

**Table 9:** The role of dietary *β*-carotene supplementation in the oriental river prawn: Impacts on intestinal microflora following an 8-week feeding trial.

Parameters	*β*-Carotene (mg/kg)					One- way ANOVA		
	Control	50	100	150	200	*F*	d.f.	*p*-Value
Total bacterial count (TBC; log_10_ CFU/g)	7.41 ± 0.19^a^	7.24 ± 0.14^ab^	7.03 ± 0.23^bc^	6.78 ± 0.09^cd^	6.59 ± 0.07^d^	18.193	4	0.001
Lactic acid bacteria (LAB; log_10_ CFU/g)	1.16 ± 0.03	1.16 ± 0.02	1.18 ± 0.03	1.16 ± 0.02	1.20 ± 0.04	3.017	4	0.142

*Note:* Values are presented as mean ± standard deviation (M ± SD, *n* = 3). A significant difference between the treatments is indicated by means in the same row with different superscript letters (*p* < 0.05).

**Table 10:** The role of dietary *β*-carotene supplementation in the oriental river prawn: Impacts on total carotenoid content (TCC) following an 8-week feeding trial.

Parameters	*β*-Carotene (mg/kg)					One- way ANOVA		
	Control	50	100	150	200	*F*	d.f.	*p*-Value
Muscle (μg/g)	2.32 ± 0.19^d^	17.22 ± 1.92^c^	20.15 ± 1.36^b^	21.62 ± 1.44^b^	23.41 ± 1.64^a^	90.479	4	0.001
Shell (μg/g)	5.89 ± 0.36^e^	35.72 ± 2.55^d^	62.32 ± 5.12^c^	69.81 ± 3.76^b^	75.27 ± 4.83^a^	239.722	4	0.001
Hepatopancreas (μg/g)	3.69 ± 0.76^e^	20.13 ± 1.48^d^	27.11 ± 2.30^c^	36.41 ± 2.19^b^	43.66 ± 3.18^a^	133.029	4	0.001

*Note:* Values are presented as mean ± standard deviation (M ± SD, *n* = 3). A significant difference between the treatments is indicated by means in the same row with different superscript letters (*p* < 0.05).

**Table 11:** The role of dietary *β*-carotene supplementation in the oriental river prawn: Impacts on whole-body proximate composition following an 8-week feeding trial.

Parameters	*β*-Carotene (mg/kg)					One- way ANOVA		
	Control	50	100	150	200	*F*	d.f.	*p*-Value
Moisture (%)	75.16 ± 0.52^a^	74.61 ± 0.38^b^	74.33 ± 0.24^b^	72.82 ± 0.20^c^	72.42 ± 0.33^c^	65.446	4	0.001
Crude protein (%)	15.46 ± 0.29^c^	16.37 ± 0.22^b^	16.59 ± 0.31^b^	17.66 ± 0.15^a^	17.96 ± 0.42^a^	52.992	4	0.001
Crude lipid (%)	2.58 ± 0.40^c^	3.92 ± 0.09^b^	4.41 ± 0.18^a^	4.36 ± 0.17^a^	4.45 ± 0.11^a^	48.312	4	0.001
Ash (%)	5.78 ± 0.31	5.94 ± 0.23	5.82 ± 0.19	5.75 ± 0.12	5.89 ± 0.08	0.024	4	0.993

*Note:* Values are presented as mean ± standard deviation (M ± SD, *n* = 3). A significant difference between the treatments is indicated by means in the same row with different superscript letters (*p* < 0.05).

**Table 12:** The role of dietary *β*-carotene supplementation in the oriental river prawn: Impacts on muscle tissue amino acid profiles (% dry matter) following an 8-week feeding trial.

Parameters	*β*-Carotene (mg/kg)					One- way ANOVA		
	Control	50	100	150	200	*F*	d.f.	*p*-Value
EAAs								
Arginine	6.41 ± 0.03^c^	6.56 ± 0.09^bc^	6.69 ± 0.12^b^	6.85 ± 0.05^a^	6.97 ± 0.11^a^	19.650	4	0.001
Histidine	2.32 ± 0.02^d^	2.39 ± 0.06^cd^	2.47 ± 0.04^bc^	2.51 ± 0.09^b^	2.62 ± 0.05^a^	12.754	4	0.001
Isoleucine	3.68 ± 0.05^c^	3.73 ± 0.02^c^	3.88 ± 0.05^b^	4.01 ± 0.06^a^	3.98 ± 0.03^a^	37.617	4	0.001
Leucine	5.05 ± 0.09^b^	5.12 ± 0.05^b^	5.37 ± 0.11^a^	5.31 ± 0.15^a^	5.44 ± 0.04^a^	8.561	4	0.003
Lysine	5.91 ± 0.11^c^	6.19 ± 0.04^b^	6.22 ± 0.06^b^	6.30 ± 0.05^ab^	6.39 ± 0.08^a^	19.085	4	0.001
Methionine	2.17 ± 0.03^c^	1.97 ± 0.02^d^	2.24 ± 0.07^bc^	2.33 ± 0.05^a^	2.30 ± 0.02^ab^	33.907	4	0.001
Phenylalanine	3.43 ± 0.07^c^	3.51 ± 0.03^bc^	3.56 ± 0.04^b^	3.62 ± 0.10^ab^	3.71 ± 0.05^a^	8.540	4	0.003
Threonine	3.01 ± 0.04^c^	3.15 ± 0.09^b^	3.27 ± 0.06^a^	3.23 ± 0.05^ab^	3.34 ± 0.06^a^	12.371	4	0.001
Tryptophan	0.77 ± 0.02^c^	0.81 ± 0.02^b^	0.84 ± 0.01^b^	0.89 ± 0.03^a^	0.92 ± 0.02^a^	24.750	4	0.001
Valine	3.51 ± 0.07^d^	3.73 ± 0.05^c^	3.89 ± 0.07^b^	4.05 ± 0.02^a^	4.13 ± 0.06^a^	57.350	4	0.001
* Σ*EAA	36.26 ± 0.82^c^	37.16 ± 0.73^c^	38.43 ± 0.29^b^	39.10 ± 0.61^ab^	39.98 ± 0.55^a^	16.947	4	0.001
NEAAs								
Alanine	4.58 ± 0.05^b^	4.66 ± 0.03^b^	4.71 ± 0.11^b^	4.85 ± 0.04^a^	4.91 ± 0.08^a^	11.789	4	0.001
Aspartic acid	8.07 ± 0.14	7.83 ± 0.10	7.85 ± 0.06	7.79 ± 0.16	7.75 ± 0.12	3.180	4	0.063
Cysteine	1.63 ± 0.05	1.53 ± 0.02	1.59 ± 0.05	1.61 ± 0.07	1.60 ± 0.03	1.902	4	0.187
Glutamic acid	11.53 ± 0.09	11.66 ± 0.16	11.49 ± 0.24	11.58 ± 0.12	11.51 ± 0.21	0.450	4	0.770
Glycine	5.72 ± 0.12^a^	5.53 ± 0.04^b^	5.47 ± 0.06^b^	5.45 ± 0.09^b^	5.41 ± 0.11^b^	5.608	4	0.012
Proline	3.15 ± 0.07^a^	3.05 ± 0.04^a^	3.11 ± 0.08^a^	2.93 ± 0.05^b^	2.87 ± 0.04^b^	12.507	4	0.001
Serine	3.54 ± 0.03	3.56 ± 0.05	3.52 ± 0.02	3.59 ± 0.03	3.55 ± 0.03	1.795	4	0.207
Tyrosine	3.18 ± 0.05^a^	3.14 ± 0.07^a^	3.09 ± 0.05^ab^	2.97 ± 0.03^c^	3.01 ± 0.02^bc^	10.214	4	0.001
* Σ*NEAA	41.40 ± 0.79	40.96 ± 0.93	40.83 ± 0.56	40.77 ± 1.02	40.61 ± 0.68	0.407	4	0.800
* Σ*EAA/*Σ*NEAA	0.88 ± 0.01^d^	0.91 ± 0.01^c^	0.94 ± 0.01^b^	0.96 ± 0.02^ab^	0.98 ± 0.01^a^	29.625	4	0.001

*Note:* Values are presented as mean ± standard deviation (M ± SD, *n* = 3). A significant difference between the treatments is indicated by means in the same row with different superscript letters (*p* < 0.05).

Abbreviations: EAA, essential amino acids; NEAA, non-EAAs.

**Table 13:** The role of dietary *β*-carotene supplementation in the oriental river prawn: Impacts on muscle tissue fatty acid compositions (% dry matter) following an 8-week feeding trial.

Parameters	*β*-Carotene (mg/kg)					One- way ANOVA		
	Control	50	100	150	200	*F*	d.f.	*p*-Value
C14:0	3.83 ± 0.03^a^	3.81 ± 0.02^ab^	3.78 ± 0.02^bc^	3.75 ± 0.02^c^	3.68 ± 0.03^d^	17.250	4	0.001
C15:0	0.49 ± 0.01^a^	0.48 ± 0.02^a^	0.44 ± 0.01^b^	0.46 ± 0.02^ab^	0.40 ± 0.02^c^	13.714	4	0.001
C16:0	16.67 ± 0.06^a^	15.66 ± 0.03^b^	14.37 ± 0.02^c^	13.10 ± 0.03^d^	11.61 ± 0.04^e^	818.034	4	0.001
C17:0	0.29 ± 0.01^a^	0.26 ± 0.01^b^	0.26 ± 0.02^b^	0.23 ± 0.01^c^	0.21 ± 0.01^c^	17.813	4	0.001
C18:0	4.48 ± 0.02^d^	4.52 ± 0.02^c^	4.58 ± 0.01^b^	4.65 ± 0.01^a^	4.62 ± 0.02^a^	52.500	4	0.001
C20:0	0.43 ± 0.01^a^	0.39 ± 0.01^b^	0.41 ± 0.02^ab^	0.41 ± 0.01^ab^	0.39 ± 0.01^b^	5.261	4	0.015
C22:0	0.19 ± 0.01^a^	0.16 ± 0.02^bc^	0.18 ± 0.01^ab^	0.15 ± 0.02^cd^	0.13 ± 0.01^d^	7.773	4	0.004
C23:0	0.35 ± 0.02^c^	0.38 ± 0.01^b^	0.34 ± 0.01^c^	0.39 ± 0.01^b^	0.42 ± 0.02^a^	14.05	4	0.001
*Σ*SFA	26.73 ± 0.06^a^	25.66 ± 0.05^b^	24.36 ± 0.06^c^	23.14 ± 0.03^d^	21.46 ± 0.04^e^	950.537	4	0.001
C16:*n*	8.69 ± 0.03^e^	9.02 ± 0.07^d^	9.14 ± 0.02^c^	9.31 ± 0.05^b^	9.47 ± 0.06^a^	113.786	4	0.001
C17:*n*	0.38 ± 0.01^c^	0.40 ± 0.02^bc^	0.43 ± 0.01^ab^	0.46 ± 0.02^a^	0.45 ± 0.02^a^	12.107	4	0.001
C18:*n*9	33.82 ± 0.10^d^	33.94 ± 0.12^d^	34.61 ± 0.07^c^	35.09 ± 0.05^b^	35.73 ± 0.15^a^	176.373	4	0.001
C20:*n*9	0.93 ± 0.01^d^	0.97 ± 0.01^c^	1.04 ± 0.01^b^	1.02 ± 0.01^b^	1.17 ± 0.02^a^	156.188	4	0.001
C22:*n*9	0.23 ± 0.01^d^	0.27 ± 0.01^c^	0.27 ± 0.01^c^	0.29 ± 0.01^b^	0.31 ± 0.01^a^	26.400	4	0.001
*Σ*MUFA	44.05 ± 0.13^e^	44.60 ± 0.11^d^	45.49 ± 0.07^c^	46.17 ± 0.21^b^	47.13 ± 0.05^a^	280.327	4	0.001
C18:2*n*6	19.37 ± 0.02^d^	19.46 ± 0.01^cd^	19.52 ± 0.04^bc^	19.61 ± 0.011^b^	19.90 ± 0.08^a^	30.051	4	0.001
C18:3*n*6	0.52 ± 0.01^e^	0.59 ± 0.02^d^	0.62 ± 0.02^c^	0.71 ± 0.01^b^	0.79 ± 0.01^a^	151.773	4	0.001
C18:3*n*3	1.66 ± 0.01^d^	1.69 ± 0.01^c^	1.73 ± 0.01^b^	1.80 ± 0.01^a^	1.81 ± 0.01^a^	131.100	4	0.001
C20:2*n*	2.47 ± 0.02^d^	2.53 ± 0.01^c^	2.55 ± 0.01^c^	2.62 ± 0.02^b^	2.68 ± 0.02^a^	71.250	4	0.001
C20:3*n*3	0.56 ± 0.01^c^	0.57 ± 0.01^c^	0.61 ± 0.01^b^	0.63 ± 0.01^a^	0.62 ± 0.01^ab^	29.100	4	0.001
C20:5*n*3 (EPA)	2.20 ± 0.01^e^	2.29 ± 0.01^d^	2.34 ± 0.01^c^	2.41 ± 0.01^b^	2.55 ± 0.01^a^	521.100	4	0.001
C22:5*n*3 (DPA)	0.31 ± 0.01^d^	0.34 ± 0.01^c^	0.39 ± 0.01^b^	0.44 ± 0.01^ab^	0.47 ± 0.01^a^	31.440	4	0.001
C22:6*n*3 (DHA)	2.11 ± 0.02^e^	2.23 ± 0.03^d^	2.35 ± 0.02^c^	2.44 ± 0.01^b^	2.57 ± 0.03^a^	177.778	4	0.001
*Σ*PUFA	29.20 ± 0.14^e^	29.71 ± 0.09^d^	30.11 ± 0.05^c^	30.66 ± 0.11^b^	31.39 ± 0.07^a^	228.410	4	0.001

*Note:* Values are presented as mean ± standard deviation (M ± SD, *n* = 3). A significant difference between the treatments is indicated by means in the same row with different superscript letters (*p* < 0.05).

Abbreviations: DHA, docosahexaenoic acid; DPA, docosapentaenoic acid; EPA, eicosapentaenoic acid; MUFA, monounsaturated fatty acid; PUFA, polyunsaturated fatty acid; SFA, saturated fatty acid.

## Data Availability

The data will be made available on request.
